# Interactive effects of MnO_2_, organic matter and pH on abiotic formation of N_2_O from hydroxylamine in artificial soil mixtures

**DOI:** 10.1038/srep39590

**Published:** 2017-02-01

**Authors:** Shurong Liu, Anne E. Berns, Harry Vereecken, Di Wu, Nicolas Brüggemann

**Affiliations:** 1 Institute of Bio- and Geosciences – Agrosphere (IBG-3), Forschungszentrum Jülich GmbH, 52425, Jülich, Germany

## Abstract

Abiotic conversion of the reactive nitrification intermediate hydroxylamine (NH_2_OH) to nitrous oxide (N_2_O) is a possible mechanism of N_2_O formation during nitrification. Previous research has demonstrated that manganese dioxide (MnO_2_) and organic matter (OM) content of soil as well as soil pH are important control variables of N_2_O formation in the soil. But until now, their combined effect on abiotic N_2_O formation from NH_2_OH has not been quantified. Here, we present results from a full-factorial experiment with artificial soil mixtures at five different levels of pH, MnO_2_ and OM, respectively, and quantified the interactive effects of the three variables on the NH_2_OH-to-N_2_O conversion ratio (R_NH2OH-to-N2O_). Furthermore, the effect of OM quality on R_NH2OH-to-N2O_ was determined by the addition of four different organic materials with different C/N ratios to the artificial soil mixtures. The experiments revealed a strong interactive effect of soil pH, MnO_2_ and OM on R_NH2OH-to-N2O_. In general, increasing MnO_2_ and decreasing pH increased R_NH2OH-to-N2O_, while increasing OM content was associated with a decrease in R_NH2OH-to-N2O_. Organic matter quality also affected R_NH2OH-to-N2O_. However, this effect was not a function of C/N ratio, but was rather related to differences in the dominating functional groups between the different organic materials.

Nitrous oxide (N_2_O) is a potent greenhouse gas that can be formed by several soil processes, such as microbial nitrification and denitrification. The N_2_O production from nitrification, especially from its reactive intermediate hydroxylamine (NH_2_OH), has received increasing attention in the recent past, fostered by the development of analytical techniques for the determination of the ^15^N site preference in the N_2_O molecule that allows for constraining the contribution of different source processes to total N_2_O formation^1^,^2^,^3^,^4^. Also, increasing knowledge from molecular biological and genetic studies has contributed to elucidating the different N_2_O formation mechanisms during nitrification^3^. Still, the role of NH_2_OH in N_2_O formation in the soil is insufficiently understood. While there is evidence, e.g., from measurements in wastewater treatment systems that NH_2_OH can contribute about 65% of total N_2_O formation^2^, the formation of N_2_O from NH_2_OH in soil and its controlling factors have rarely been studied^5^,^6^.

Hydroxylamine was first identified by Lees (1952)^7^ as an intermediate of the first step of nitrification by ammonia oxidizing bacteria (AOB), in which ammonia is oxidized to nitrite. Understanding the nitrification process in ammonia-oxidizing archaea (AOA), however, is much more fragmentary, but NH_2_OH has been identified as an intermediate of ammonia oxidation also in AOA^8^. In most circumstances, NH_2_OH is quickly oxidized to nitrite in the periplasm of the AOB, and N_2_O may be produced as a side product during this process^3^. However, also a leakage of NH_2_OH from the periplasm across the outer membrane of the AOB into the soil matrix, followed by a chemical reaction with soil constituents yielding N_2_O, could be a potential mechanism of N_2_O formation during nitrification. This assumption is supported by the fact that AOB can take up NH_2_OH from the surrounding medium^9^ as well as by the observation that the medium of AOB cultures contains measurable amounts of NH_2_OH. The latter was found for *Nitrosomonas europaea* under oxic conditions, both for wild-type *N. europaea* and even more so for NirK and NorB-deficient mutants^10^. In accordance with this assumption, a positive relationship between NH_2_OH content of the soil and soil N_2_O emissions under oxic conditions has been detected in natural forest soil samples^11^. In addition, abiotic formation of N_2_O from NH_2_OH has been observed in sterilized soil samples from different ecosystems^6^.

In soil, N_2_O can be formed chemically, among a range of possible reactions, according to the following equations^12^:


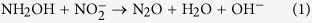



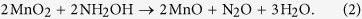


Owing to its high oxidization potential, manganese dioxide (MnO_2_) acts as a strong oxidant in soil that plays an important role not only in the turnover of organic substances^13^,^14^, but also in the N cycle^15^, even under anoxic conditions^16^,^17^. Soil organic matter (SOM) plays a crucial role in the storage and release of N as well as in the emission of N_2_O from soils. Quick disappearance of nitrite and nitrate within a few hours after addition has been observed in forest soils^18^,^19^,^20^, whereas NH_2_OH disappeared completely in soil several minutes after addition^5^,^11^. Abiotic reactions of SOM and inorganic N may contribute to the quick disappearance, as nitrite and nitrate can react with SOM or dissolved organic carbon (DOC), leading to the formation of organic N, such as nitroso and nitro compounds^21^,^22^, while NH_2_OH can also react with carbonyl groups to form oximes^23^,^24^:


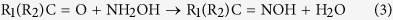


The quality of SOM, or more specifically the C/N ratio and the type and abundance of functional groups, influence the bonding of inorganic N to SOM^22^. Phenolic lignin derivatives, an important constituent of SOM, can covalently bind reactive N compounds and thereby stabilize N in soil^25^,^26^. The N binding form can be affected by the plant species from which the SOM is derived due to the different characteristics of phenolic compounds, e.g. condensed or hydrozable tannin^27^.

Soil pH is another key factor influencing most nitrogen transformations in soil. High pH can lead to an increase of chemical N_2_O production involving nitrite by favoring nitrite accumulation, either directly through increasing nitrite stability, or indirectly by inhibiting biological nitrite oxidation due to a higher concentration of free NH_3_ (an inhibitor of nitrite oxidizers) in the soil^28^. In contrast, high soil N_2_O emissions have also been observed in acid forest soils^29^,^30^. In this case, the effect of pH on enzyme activities during denitrification and nitrification was suggested as the main reason^31^. However, also chemical reactions that produce N_2_O in the soil, such as the reaction of nitrite with SOM and the reaction of NH_2_OH with MnO_2_, are subject to a strong pH dependence and can contribute substantially to N_2_O emissions under acidic conditions^32^,^33^,^34^.

The aim of this study was to quantify the interactive effects of the major control factors of abiotic N_2_O formation from NH_2_OH in soil, i.e. MnO_2_ content, pH and OM quantity and quality, by means of experiments with artificial soil mixtures. We hypothesized that the control factors interact with each other in the following way: At higher pH, unprotonated NH_2_OH would react more readily with carbonyl groups of OM, leading to oxime formation and making NH_2_OH less available for oxidation to N_2_O by MnO_2_. Lower soil pH would lead to increased protonation of NH_2_OH, making NH_2_OH more stable against the reaction with carbonyl groups of OM and more prone to the reaction with MnO_2_, leading to higher N_2_O formation from the same amount of NH_2_OH (Fig. 1). To test these hypotheses, we performed two laboratory experiments with artificial soil mixtures, which were produced from pure quartz sand, quartz powder, kaolin clay, MnO_2_ powder and different plant-derived organic materials, resembling SOM of different quality, at different mixing ratios. In these experiments, N_2_O formation was determined after NH_2_OH addition to the different mixtures at different pH levels and related to the different control factors.

## Results and Discussion

### R_NH2OH-to-N2O_ at different pH, MnO_2_ and OM contents (%)

**Figure 2:**
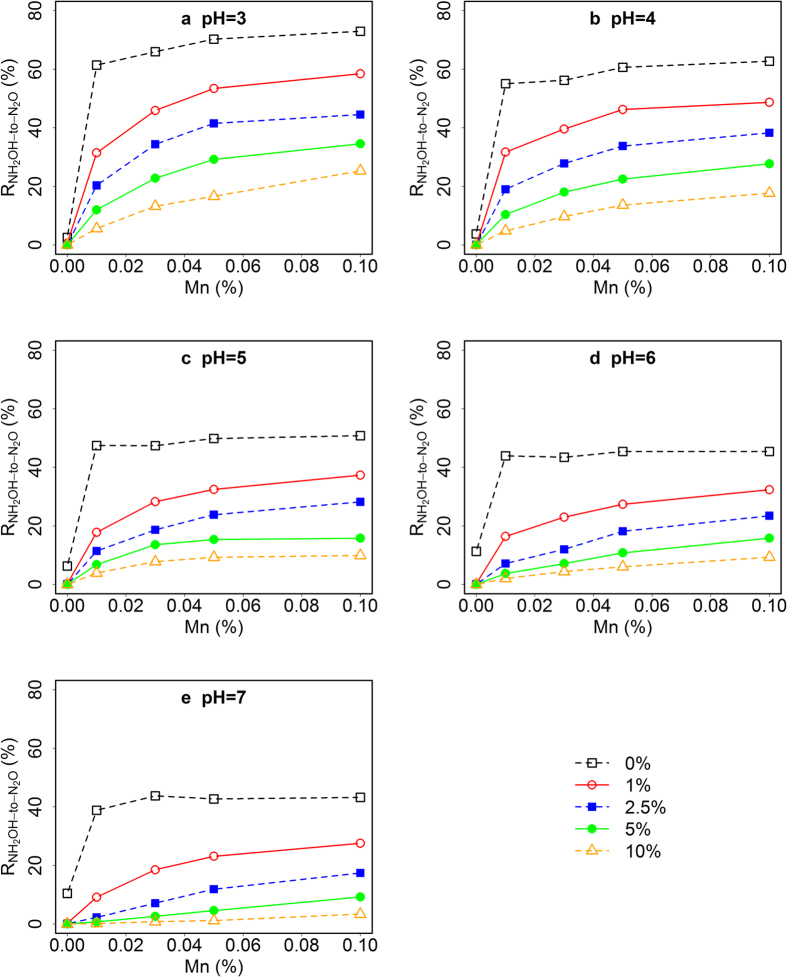
Mean NH_2_OH-to-N_2_O conversion ratios (R_NH2OH-to-N2O_) in artificial soil mixtures at different pH as well as MnO_2_ and organic matter (OM, peat moss) contents. The total amount of NH_2_OH added was 5 nmol (equivalent to 70 μg N per kg dry material). Different symbols represent R_NH2OH-to-N2O_ at different OM content (n = 3, SD < 5%, not shown).

In the present study all three factors, i.e. pH, MnO_2_ and OM content, affected R_NH2OH-to-N2O_ from peat moss significantly (Fig. 2, S1 and S2). The R_NH2OH-to-N2O_ increased greatly with an increase in MnO_2_ content from 0% to 0.1% (Fig. 2). This finding is consistent with Bremner *et al*.^5^, who studied 19 soils with a wide range of properties and found that the formation of N_2_O by decomposition of NH_2_OH was highly correlated with oxidized Mn content of the soils. The fact that NH_2_OH was used in the past for the selective extraction of Mn oxides from soil samples^35^ indicates that NH_2_OH can efficiently reduce Mn(IV) to Mn(II) or Mn(III) (and in turn is oxidized to N_2_O) in natural soil samples. With increasing OM content, R_NH2OH-to-N2O_ decreased remarkably, especially at high pH (Fig. 2c,d,e). For example, an increase in OM by only 1% at 0.01% MnO_2_ led to about 50% and 80% decrease in N_2_O emissions at pH 3 and pH 7, respectively (Fig. 2e, S2). This could be caused by the oxime-forming reaction between NH_2_OH and carbonyl groups of OM, such as in quinones. The oximes may undergo a tautomeric equilibrium with their corresponding nitrosophenol forms^23^. In fact, NH_2_OH has been used in a number of previous studies to determine the carbonyl content of humic substances^36^, indicating a high affinity of NH_2_OH to OM that contains carbonyl groups. In the absence of OM and MnO_2_, increasing pH led to a slight increase in R_NH2OH-to-N2O_ due to the self-decomposition of NH_2_OH at higher pH, whereas in the presence of OM and absence of MnO_2_ nearly no NH_2_OH was converted to N_2_O (Fig. S2a). In contrast, the effect of increasing pH on R_NH2OH-to-N2O_ became negative already in the presence of 0.01% MnO_2_ (Fig. S2b). This finding suggests that acidic conditions are favorable for the redox reaction between NH_2_OH and MnO_2_.

Also strong interactive effects of pH and MnO_2_, pH and OM, and OM and MnO_2_ were observed for the conversion of NH_2_OH to N_2_O. The largest R_NH2OH-to-N2O_ found in the present experiment was 81.5% in the absence of SOM at pH 3, and with a MnO_2_ content of 0.1% (Fig. 2a), while the lowest R_NH2OH-to-N2O_ was about 9%, when SOM content was 10% in the presence of 0.1% MnO_2_ at pH 7 (Fig. 2e). This suggests that even at the highest MnO_2_ level and in all other respects optimal conditions a small fraction of NH_2_OH had not been converted to N_2_O, but to some other unidentified product.

In the treatments without OM, MnO_2_ had only a small effect on R_NH2OH-to-N2O_ at all pH conditions, while it had a larger effect especially at higher OM content (Fig. 2, S1), suggesting a strong competition between OM and MnO_2_ for NH_2_OH. The competition was biased by pH, with lower pH favouring the reaction of NH_2_OH and MnO_2_, while higher pH favoured the reaction of NH_2_OH with OM. These findings confirmed our hypothesis that at low pH NH_2_OH is more protected against reaction with OM and more available for the oxidation by MnO_2_ due to the higher degree of NH_2_OH protonation at lower pH.

### R_NH2OH-to-N2O_ as a function of pH, MnO_2_ content and OM quality

**Figure 3:**
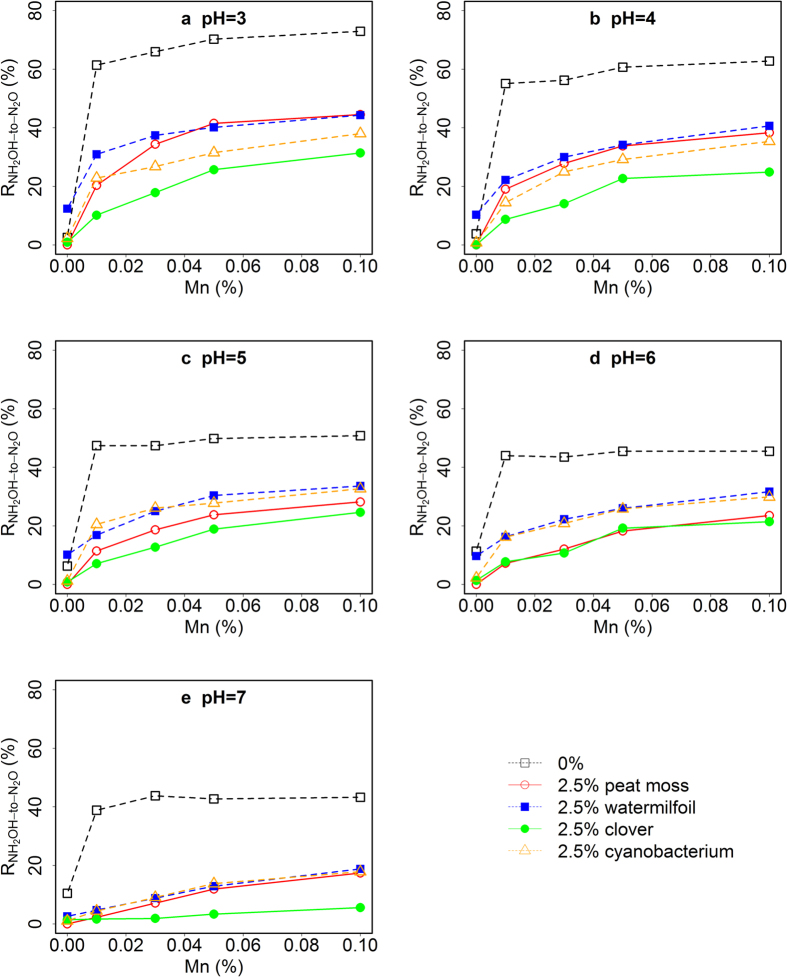
Mean NH_2_OH-to-N_2_O conversion ratios (R_NH2OH-to-N2O_) in artificial soils at different pH and MnO_2_ content, and for organic matter of different origins at a fixed content of 2.5% (w/w). The total amount of NH_2_OH added was 5 nmol. Different symbols represent R_NH2OH-to-N2O_ for the artificial soil mixtures with the different organic materials (n = 3, SD < 5%, not shown).

Organic matter quality had a clear influence on R_NH2OH-to-N2O_ in this study (Fig. 3, S3, and S4). Most of the OM types were associated with a significantly lower R_NH2OH-to-N2O_ compared to the mixtures without OM within the pH range of the experiment. In general, the inhibitory effect of the organic materials on the conversion of NH_2_OH to N_2_O showed a clear pH dependency, but was not a function of C/N ratio (Fig. 3, S3). At acidic conditions (pH 3–4), peat moss and watermilfoil with their relatively large C/N ratio inhibited R_NH2OH-to-N2O_ the least, while the cyanobacterium material and clover had a stronger inhibitory effect on R_NH2OH-to-N2O_ despite their smaller C/N ratio (Fig. 3a,b). The differences between peat moss, cyanobacterium and watermilfoil material as OM became smaller at higher pH, and were no longer significant at pH 7 in the presence of 0.01% MnO_2_ (Fig. 3e), while clover showed always the smallest R_NH2OH-to-N2O_ at all pH levels. In the absence of MnO_2_, all OM forms showed a R_NH2OH-to-N2O_ close to zero, except for the watermilfoil material that was associated with a R_NH2OH-to-N2O_ significantly above zero within the pH range 3–6 (Fig. S4a). A possible explanation could be the fact that, in contrast to the other OM sources, the watermilfoil material contained about 0.03% Mn (Table 1), which could have caused the N_2_O emission after NH_2_OH addition even without external MnO_2_ addition.

We assumed that R_NH2OH-to-N2O_ would be a function of the C/N ratio of the different SOM types, as larger C/N ratios would be indicative of a lower degree of N-containing functional groups, i.e. leaving a higher chance for NH_2_OH to react with SOM and not to be converted to N_2_O. However, as stated above we did not observe any clear relationship between C/N ratio and R_NH2OH-to-N2O_, e.g. peat moss had the largest C/N ratio, but did not lead to the lowest R_NH2OH-to-N2O_. Instead, clover with a much lower C/N ratio had the largest inhibitory effect on R_NH2OH-to-N2O._ The addition of 2.5% dry clover powder (C/N ratio = 11.3) to the artificial soil mixture decreased R_NH2OH-to-N2O_ by 48% at pH 3 (Fig. 3a), which was similar to the effect of 10% peat moss (C/N ratio = 67.2) at the same pH (Fig. 2a). The reason for this observation could lie in the differences in functional groups between the different organic materials used in this study.

**Figure 4:**
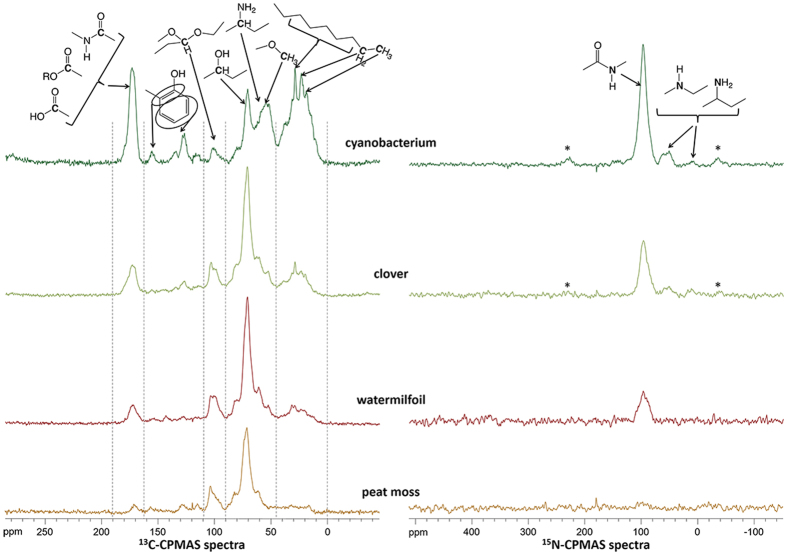
^13^C- and ^15^N-CPMAS-NMR spectra of the different organic materials (cyanobacterium, clover, watermilfoil, peat moss) used in the experiment.

A better insight into the effects of C and N functional groups of the different organic materials was obtained from NMR analysis. The peat moss OM had the lowest proportion of ester or amide carbonyl at around 170 ppm of all materials (Fig. 4, Table 2). This is in accordance with the observation that – despite having the largest C/N ratio – peat moss OM had a lower inhibitory effect on R_NH2OH-to-N2O_ compared to clover and watermilfoil OM (if the background MnO_2_ effect was subtracted), i.e. the lack of almost any carbonyl groups in peat moss was clearly visible in its chemical behaviour toward NH_2_OH. In addition, peat moss OM exhibited the largest proportion of O-substituted aliphatic compounds, which might have also contributed to the relatively low inhibitory effect on R_NH2OH-to-N2O_ in comparison to clover and watermilfoil OM. In contrast, cyanobacterium OM had the highest proportion of acid/amide carbonyl of all four organic materials, suggesting the highest inhibitory effect on R_NH2OH-to-N2O_ due to the competitive reaction of carbonyl groups with NH_2_OH. The clover material, however, contained lower amounts of O-substituted aliphatics and di-O-substituted C in comparison to peat moss and watermilfoil OM, which may have increased its affinity for NH_2_OH. For the proportion of unsaturated C no clear trend emerged across the different materials, suggesting that the effect of unsaturated C on R_NH2OH-to-N2O_ is of minor importance.

### Development of a stepwise multiple regression model from the artificial soil mixtures and application to natural soils

**Figure 5:**
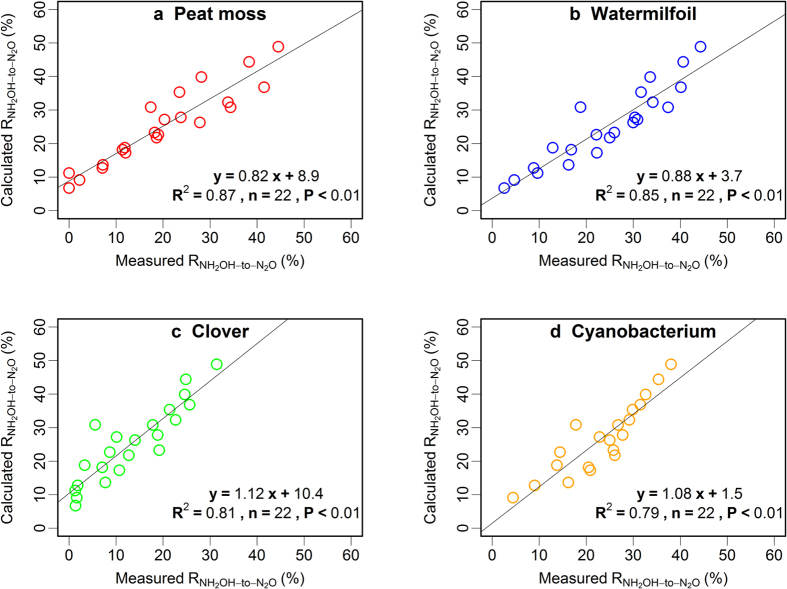
Results of the application of the artificial soil regression model for the calculation of NH_2_OH-to-N_2_O conversion ratios (R_NH2OH-to-N2O_) to artificial soil mixtures amended with the different organic materials (n = 22). The three points for which R_NH2OH-to-N2O_ was determined at pH 3, 4, and 5 without MnO_2_ addition were excluded from the simulation.

The multiple regression model obtained from the first experiments was R_NH2OH-to-N2O_ = 45.9–3.1 SOM + 241.1 MnO_2_ − 4.5 pH, R^2^ = 0.62 (*P* < 0.01), which could explain about 62% variation of R_NH2OH-to-N2O_, and the contributions of pH, Mn and SOM content to the model’s performance were all significant (*P* < 0.01). It could well explain the observations (Fig. 3) for peat moss, watermilfoil and clover OM (R^2^ close to 0.8, *P* < 0.01, Fig. 5). This demonstrated the general applicability of the model for the OM derived from the different plant and cyanobacterium materials, with different N content, aliphatic C content and C/N ratios. In contrast, the model proved to be not appropriate for the artificial soil mixture without any MnO_2_, indicated by the decreased goodness of the simulation.

**Figure 6:**
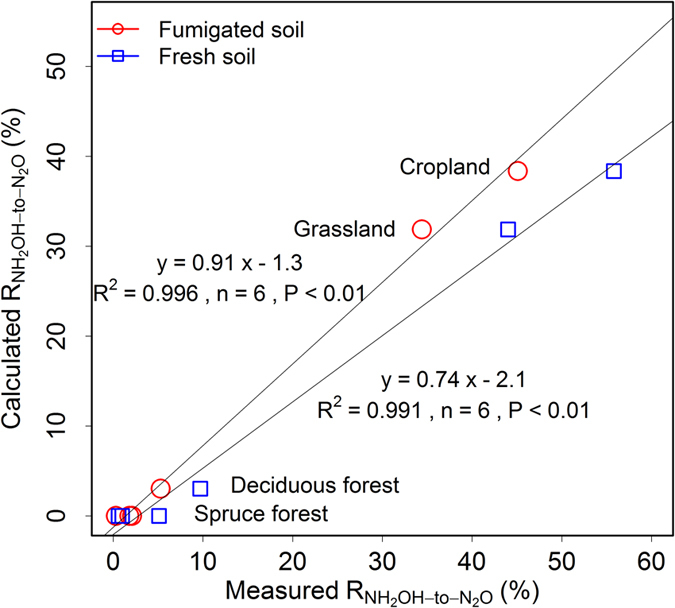
Results of the application of the artificial soil regression model for the calculation of NH_2_OH-to-N_2_O conversion ratios (R_NH2OH-to-N2O_) to six natural fresh and chloroform-fumigated soils as reported in Heil *et al*.^6^.

Finally, R_NH2OH-to-N2O_ was simulated with the same regression model for the natural soils described in Heil *et al*.^6^. The results showed that the application of the model to natural soils was promising, no matter if it was applied to fumigated or fresh soils (Fig. 6). The simulated R_NH2OH-to-N2O_ explained more than 90% of the observed rates, especially for cropland, grassland and deciduous forest soils. However, the model failed at correctly predicting R_NH2OH-to-N2O_ for the spruce forest soil of Heil *et al*.^6^, which could be related to the high SOM and relatively low MnO_2_ content of the spruce soil as compared to the other soils. This finding suggests that there is a threshold value for the SOM content of 10% above which – and a MnO_2_ content of 0.01% below which – the model fails to predict the correct R_NH2OH-to-N2O_ values.

Soil pH, MnO_2_ and SOM content were identified as crucial control variables of R_NH2OH-to-N2O_, i.e. the conversion ratio of NH_2_OH to N_2_O in the artificial soil experiments of this study. Organic matter derived from different plant species and a cyanobacterium also affected R_NH2OH-to-N2O_ due to the differences in composition, type and abundance of functional groups, as more carbonyl C leads to higher reactivity of NH_2_OH with organic matter, thereby lowering its availability for the oxidation to N_2_O by MnO_2_. The multiple regression model of pH, MnO_2_ and OM developed here could explain about 60% of the variance of R_NH2OH-to-N2O_ in the artificial soil mixtures, and proved also to be promising for the prediction of R_NH2OH-to-N2O_ of chemical N_2_O production from NH_2_OH in natural soils, when SOM content was below 10% and Mn content was larger than 0.01%. If these findings can be confirmed for other soils from different ecosystems, this improved understanding of the controls of N_2_O formation from the reactive nitrification intermediate NH_2_OH in soils can have large implications for developing appropriate management options, such as adding organic amendments with suitable chemical characteristics, for mitigating N_2_O emissions from agricultural land, the largest anthropogenic source of N_2_O to the atmosphere.

## Methods

### Experimental setup

Two full-factorial artificial soil experiments were conducted. The first experiment comprised three factors (pH, MnO_2_ and OM content) and five levels of each factor. The scond experiment comprised also three factors (pH, MnO_2_ and OM quality) with five levels of pH and MnO_2_, and four different organic materials at the same concentration level (2.5% w/w on a dry weight basis), but of different quality. Each experiment was conducted in triplicate.

### Preparation of the artificial soil mixtures

The artificial soil mixtures consisted of 15% (expressed as percentage of dry weight) fine quartz sand (50% of the particles 0.05–0.2 mm), representing the sand fraction, 65% quartz powder (0.002–0.063 mm), representing the silt fraction, and 20% kaolin clay (≤0.002 mm), representing the clay fraction, mimicking the soil texture of the agricultural Terrestrial Environmental Observatories (TERENO) field site Selhausen^37^. Freeze-dried, finely ground and sieved (<0.75 mm) peat moss (*Sphagnum magellanicum*, collected from Dürres Maar, Eifel, Germany) was amended as SOM to the artificial soil mixtures at levels of 0%, 1%, 2.5%, 5%, 10% dry weight, while the relative amount of sand, clay and silt was reduced according to the amount of peat moss added. The water holding capacity (WHC) was determined for each of the artificial soil mixtures. The WHC increased with increasing organic matter (OM) content, and amounted to 29%, 44%, 55%, 76%, and 132% for the five OM contents, respectively. Each of those artificial soil mixtures was amended with MnO_2_ (Merck, Darmstadt, Germany) at five different levels (0%, 0.01%, 0.025%, 0.05%, 0.1% Mn), then the ingredients were thoroughly homogenized.

### Preparation of artificial soil mixtures with different OM qualities

Organic materials with different C/N ratios (Table 1) were derived from two different plant species, i.e. watermilfoil (*Myriophyllum* spec.) and clover (*Trifolium repens*), and from a cyanobacterium (*Spirulina platensis*). Watermilfoil and clover had been collected previously on the campus of Forschungszentrum Jülich (2004 and 2014, respectively), while the cyanobacterium material had been purchased in 2006 (Concept Vitalprodukte, Schwerte, Nordrhein-Westfalen, Germany). The finely ground, freeze-dried and sieved (<0.75 mm) organic material was amended to the inorganic quartz-kaolin mixture as described above at a rate of 2.5% dry weight, while the relative amount of sand, clay and silt was reduced accordingly. Also for this experiment, each of the artificial soil mixtures was amended with MnO_2_ at five different levels (0%, 0.01%, 0.025%, 0.05%, 0.1% Mn), and again mixed thoroughly to obtain a homogeneous composition.

### Addition of NH_2_OH to the artificial soil mixtures and analysis of the N_2_O formed

One gram of each artificial soil mixture was weighed into individual 22-mL gas chromatograph (GC) vials. Subsequently, NH_2_OH in different buffer solutions was added to each vial to obtain a soil water content of 50% WHC, which required addition of varying volumes of buffer solution to the different soil mixtures depending on the OM content, and adaptation of the NH_2_OH concentration of each of the buffer solutions accordingly. The total amount of NH_2_OH added to each of the soil mixtures was always 5 nmol (equivalent to 70 μg N per kg dry material). The pH buffer solutions at pH 3, 4, 5 and 6 were prepared with citric acid (0.1 M) and sodium citrate (0.1 M) according to Gomori^38^, whereas the buffer at pH 7 was prepared with tris(hydroxymethyl)aminomethane and maleate (Tris-maleate buffer). The vials were closed immediately after NH_2_OH addition. After 10 hours of incubation, the N_2_O concentration in the headspace of the vials was measured with a GC equipped with an electron capture detector (Clarus 580, PerkinElmer, Rodgau, Germany). Details of the GC setup and analytical conditions have been described previously^11^.

### Calculation of the NH_2_OH-to-N_2_O conversion ratio

The NH_2_OH-to-N_2_O conversion ratio (R_NH2OH-to-N2O_, moles N_2_O-N per mole NH_2_OH-N, %) was determined according to the following equation:


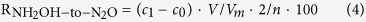


where *c*_*0*_ is the background N_2_O mixing ratio in the headspace of the control with the same amount of water instead of NH_2_OH solution (nL L^−1^); *c*_*1*_ is the N_2_O mixing ratio in the headspace of the sample with NH_2_OH addition (nL L^−1^); the factor 2 represents the molar N ratio of N_2_O and NH_2_OH; *V* is the volume of the vial headspace (0.022 L); *V*_*m*_ is the molar volume of N_2_O at standard pressure and room temperature (24.465 L mol^−1^); *n* is the amount of NH_2_OH added to the sample vials (5 nmol).

### Determination of the basic properties of the organic materials

Three replicates of each organic material were analyzed to determine its basic properties. The C and N content of the different organic materials was analyzed by weighing 200–300 μg dry material into tin capsules, followed by combustion at 1080 °C in an elemental analyzer (EuroEA, EuroVector, Milan, Italy) interfaced to an isotope-ratio mass spectrometer (Isoprime, Isoprime Ltd, Stockport, United Kingdom). The C and N content was determined through peak integration of m/z 44 (CO_2_) and 28 (N_2_), respectively, and calibrated against elemental standards.

The elemental composition of the organic materials was analyzed by using inductively coupled plasma optical emission spectrometry (ICP-OES) in the central analytical laboratory (ZEA-3) of Forschungszentrum Jülich. Briefly, 100 mg of sample material were mixed with 3 mL HNO_3_ and 2 mL H_2_O_2_, heated in the microwave at 800 W for 30 min. The mixtures were subsequently filled up to 14 mL and diluted 10-fold with deionized water followed by the ICP-OES measurement.

For the determination of characteristic molecule structures and functional groups of the different organic materials used in the experiments, ^13^C and ^15^N cross-polarisation magic-angle spinning (CPMAS) nuclear magnetic resonance (NMR) spectra were obtained. ^13^C CPMAS spectra were obtained on a 7.05 T Varian INOVA^TM^ Unity (Varian Inc., Palo Alto, CA, USA) at a ^13^C resonance frequency of 75.4 MHz. ^15^N CPMAS spectra were obtained on a 14.09 T Varian NMR system (Varian Inc., Palo Alto, CA, USA) at a ^15^N resonance frequency of 60.8 MHz. Samples were packed into 6 mm diameter cylindrical zirconia rotors with Vespel^®^ drive tips and spun at 8000 ± 3 Hz in an HX Apex probe. The spectra were collected with a sweep width of 25 kHz and an acquisition time of 20 ms. In preliminary experiments, the optimal contact time and recycle delay for the cross-polarization experiment were determined. A contact time of 1 ms and a 5 s recycle delay time were used for ^13^C, whereas a contact time of 1 ms and a 1 s recycle delay time were used for ^15^N. During cross-polarization the ^1^H radio frequency (RF) field strength was set to 47 kHz for ^13^C and to 33.7 kHz for ^15^N, respectively. The ^13^C and ^15^N RF field strength was set to 41 and 41.7 kHz, respectively. An ascending ramp of 15 and 12.2 kHz on the ^1^H-RF field was used for ^13^C and ^15^N during contact time to account for inhomogeneities of the Hartmann-Hahn condition, respectively^39^. Proton decoupling was done using a spinal sequence with a ^1^H field strength of 50 and 35.6 kHz, a phase of 4.5° and 5.5°, and a pulse length of 12 and 9.5 μs, respectively.

The free induction decays (FID) were recorded with VnmrJ (Version 1.1 RevisionD, Varian Inc., Palo Alto, CA, USA) and processed with Mestre-C (Version 4.9.9.9, Mestrelab Research, Santiago de Compostela, Spain). All FIDs were fourier-transformed with an exponential filter function with a line broadening (LB) of 20 to 50 Hz. Baseline correction was done using the manual baseline correction function of Mestre-C.

The ^13^C chemical shifts are reported relative to tetramethylsilane (=0 ppm) using adamantane as an external reference. The relative intensities of the regions were determined using the integration routine of the MestRe-C software. The ^15^N chemical shifts are reported relative to ammonium nitrate (NH_4_^+^ = 0 ppm).

### Data analysis

The homogeneity of variance was tested with the Bartlett test. One-way analysis of variance (one-way ANOVA) of the main controlling factors in the two experiments was performed, followed by a Tukey Honest Significant Difference (HSD) test. A stepwise multiple regression model for the NH_2_OH-to-N_2_O conversion ratio was developed on the basis of the co-variables pH, MnO_2_ and SOM content by using the data from the first experiment. In this case, significance was tested with the F test. Linear regression was performed for simulated and measured R_NH2OH-to-N2O_ in artificial and natural soils described in Heil *et al*.^6^ and tested for significance. All analyses were performed with the R software package (version 3.1.0, R Development Core Team, 2013)^40^.

## Additional Information

**How to cite this article**: Liu, S. *et al*. Interactive effects of MnO_2_, organic matter and pH on abiotic formation of N_2_O from hydroxylamine in artificial soil mixtures. *Sci. Rep.* **6**, 39590; doi: 10.1038/srep39590 (2016).

**Publisher's note:** Springer Nature remains neutral with regard to jurisdictional claims in published maps and institutional affiliations.

## Supplementary Material

Supplementary Information

## Figures and Tables

**Figure 1:**
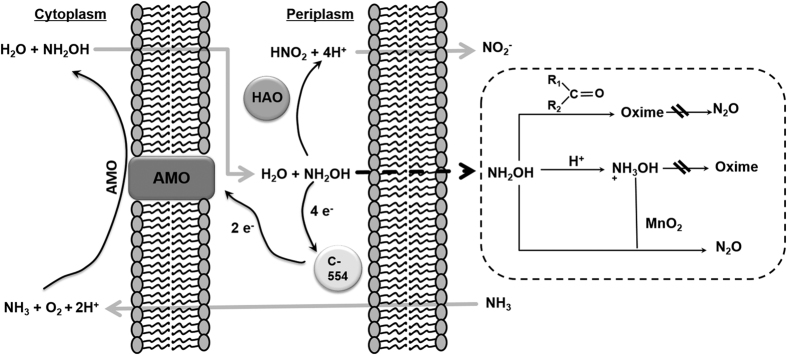
Hypothetical model of NH_2_OH release by ammonia-oxidizing bacteria to the soil environment and potential abiotic reactions of NH_2_OH with MnO_2_ and organic matter in the soil at different pH conditions (R_1_R_2_C=O represents carbonyl groups of SOM). AMO is ammonia monooxygenase; HAO is hydroxylamine oxidoreductase.

**Table 1:** Element contents (%) and C/N ratios of the organic materials used in this study.

	C	N	C/N	Al	Ca	Fe	K	Mg	Mn	Na	P	Si
Peat moss	41.3^!^	0.6	67.2	0.03	0.13	0.06	0.06	0.07	<0.01	0.01	0.03	0.08
Watermilfoil	35.4	2.1	17.0	0.12	2.26	0.11	1.21	0.25	0.031	0.67	0.12	0.21
Clover	41.4	3.7	11.3	<0.01	1.10	0.01	2.68	0.20	<0.01	<0.01	0.34	0.03
Cyanobacterium	44.9	9.9	4.5	0.02	0.31	0.09	1.22	0.31	<0.01	1.36	0.92	0.07

All elements are reported as % of dry weight (mean of three replicates). The standard deviation is 3% for the values larger than 1%, 20% for the values smaller than 0.1%, and 10% for the values in the range of 0.1% to 1%.

**Table 2:** Relative proportions of chemical features of the different plant materials derived from ^13^C CPMAS NMR spectra.

Spectral range (ppm)	Chemical features	Found in	Cyanobacterium (%)	Clover (%)	Watermilfoil (%)	Peat moss (%)
45–0	Aliphatic compounds	waxes, suberin, cutin, cyanophycin, chlorophyll (a,b,d)	41	17	15	11
64.5–45	N- and O-substituted aliphats	amino acids, amino sugars, lignin, cyanophycin	19	14	14	12
90–64.5	O-substituted aliphats	polysaccharides, cellulose, hemi-cellulose, starch, pectin, lignin	14	38	42	49
109–90	di-O-substituted C	polysaccharides, cellulose, hemi-cellulose, starch, pectin	3	11	12	14
162–109	unsaturated C, aromatic C	suberin, lignin, chlorophyll	7	11	10	11
190–162	acid, ester, amide	cutin, proteins, cyanophycin, chlorophyll	17	10	7	4

Sums within columns greater than 100 are due to rounding errors.
