# Supplementary material for: Interactive effects of MnO2, organic matter and pH on abiotic formation of N2O from hydroxylamine in artificial soil mixtures
Source: Sci Rep. 2017 Feb 1;7:39590. doi: 10.1038/srep39590 (PMC5286404; doi:10.1038/srep39590)
Supplement: Supplementary Information [file srep39590-s1.pdf]

Interactive effects of MnO_2, organic matter and pH on abiotic formation of N_2O from hydroxylamine in artificial soil mixtures

Shurong Liu, Anne E. Berns, Harry Vereecken, Di Wu, Nicolas Brüggemann

Institute of Bio- and Geosciences – Agrosphere (IBG-3), Forschungszentrum Jülich GmbH, 52425 Jülich, Germany

Supplementary Information

Supplementary figures

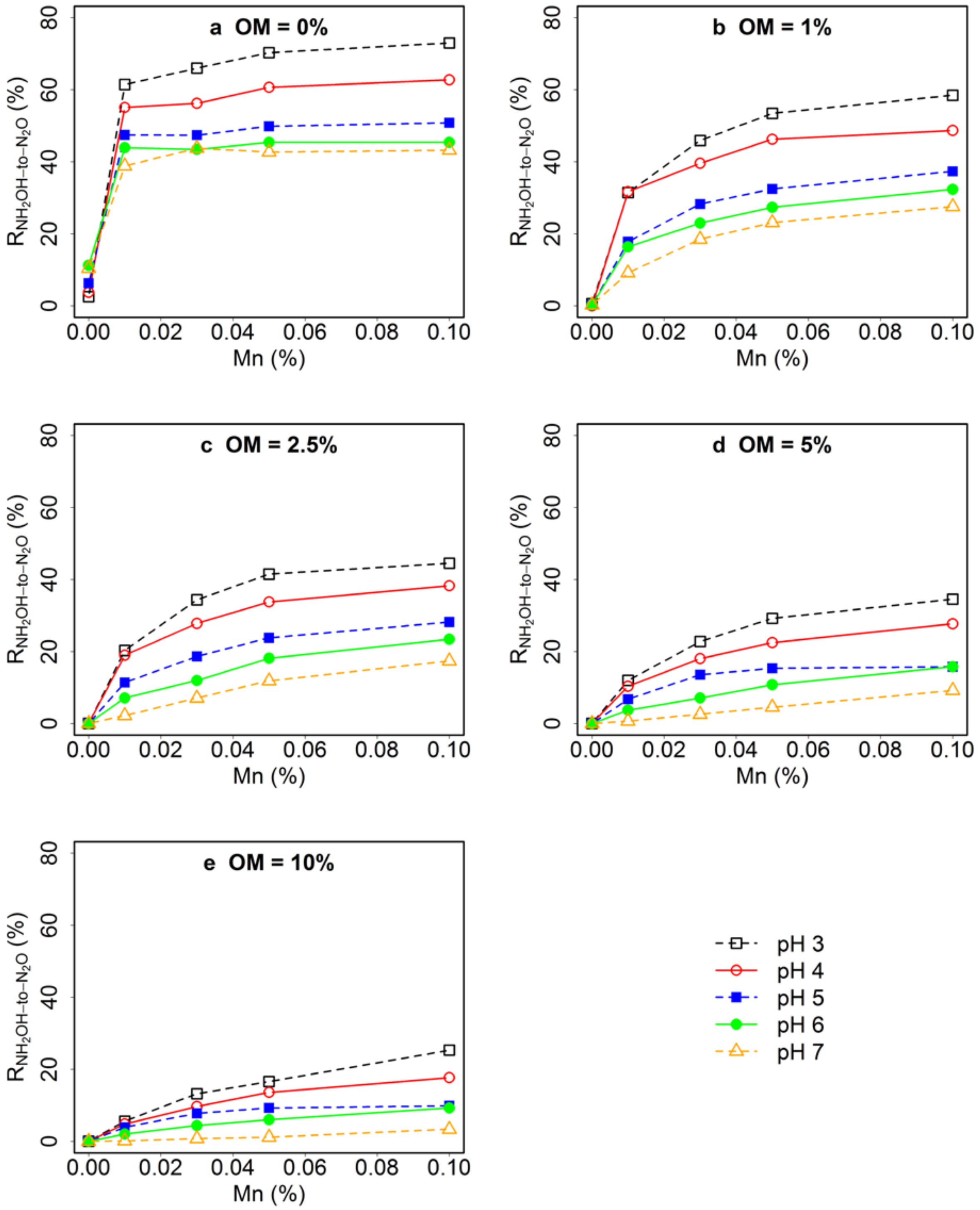

Figure S1 NH_2OH-to-N_2O conversion ratios ($R_{NH_2OH\text{-to-}N_2O}$) in artificial soil mixtures at different pH as well as MnO_2 and organic matter (OM, peat moss) contents. The total amount of NH_2OH added was 5 nmol. Different symbols represent $R_{NH_2OH\text{-to-}N_2O}$ at different pH levels.

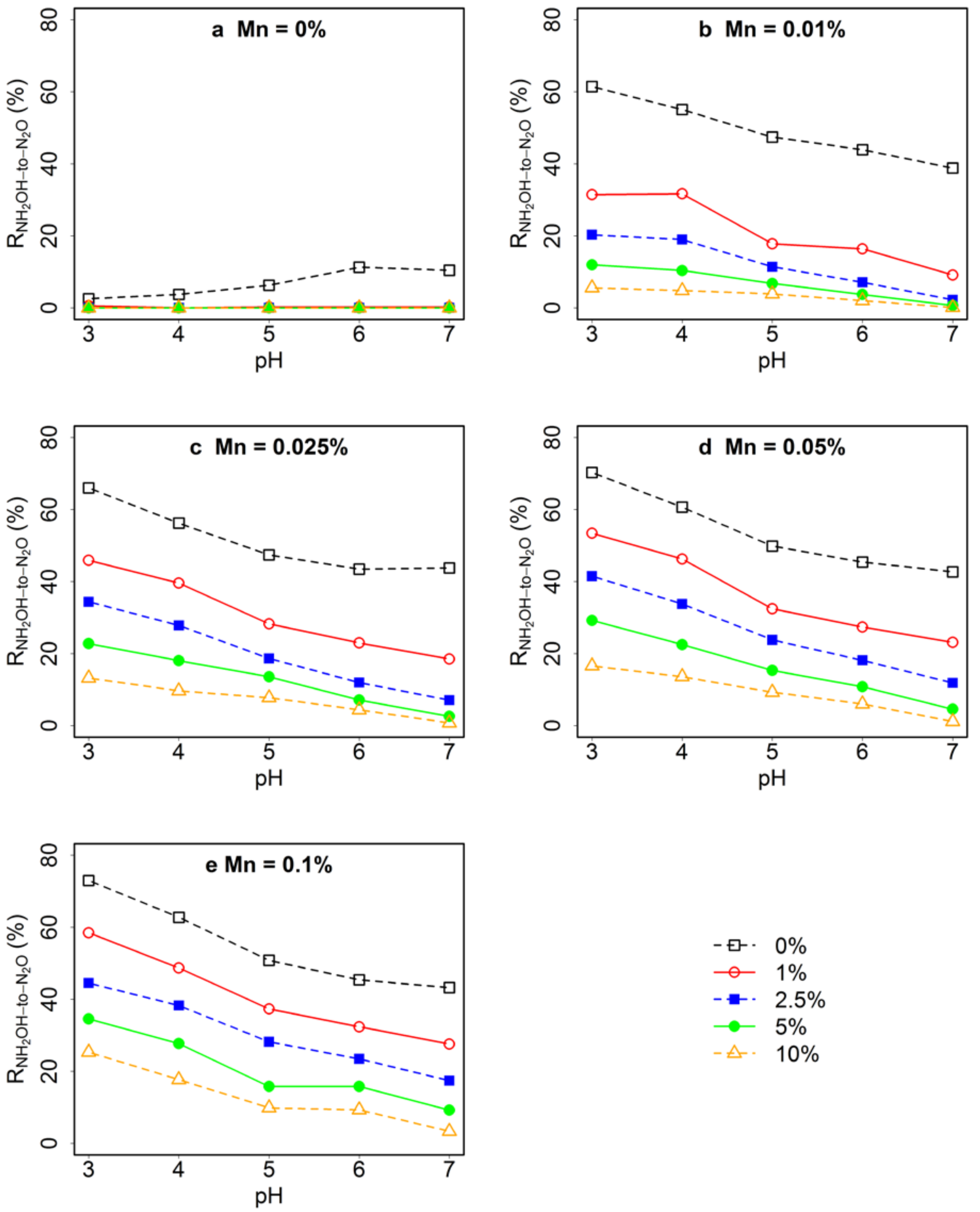

Figure S2 NH_2OH-to-N_2O conversion ratios ($R_{NH_2OH\text{-to-}N_2O}$) in artificial soil mixtures at different pH as well as MnO_2 and organic matter (OM, peat moss) contents. The total amount of NH_2OH added was 5 nmol. Different symbols represent $R_{NH_2OH\text{-to-}N_2O}$ at different OM contents.

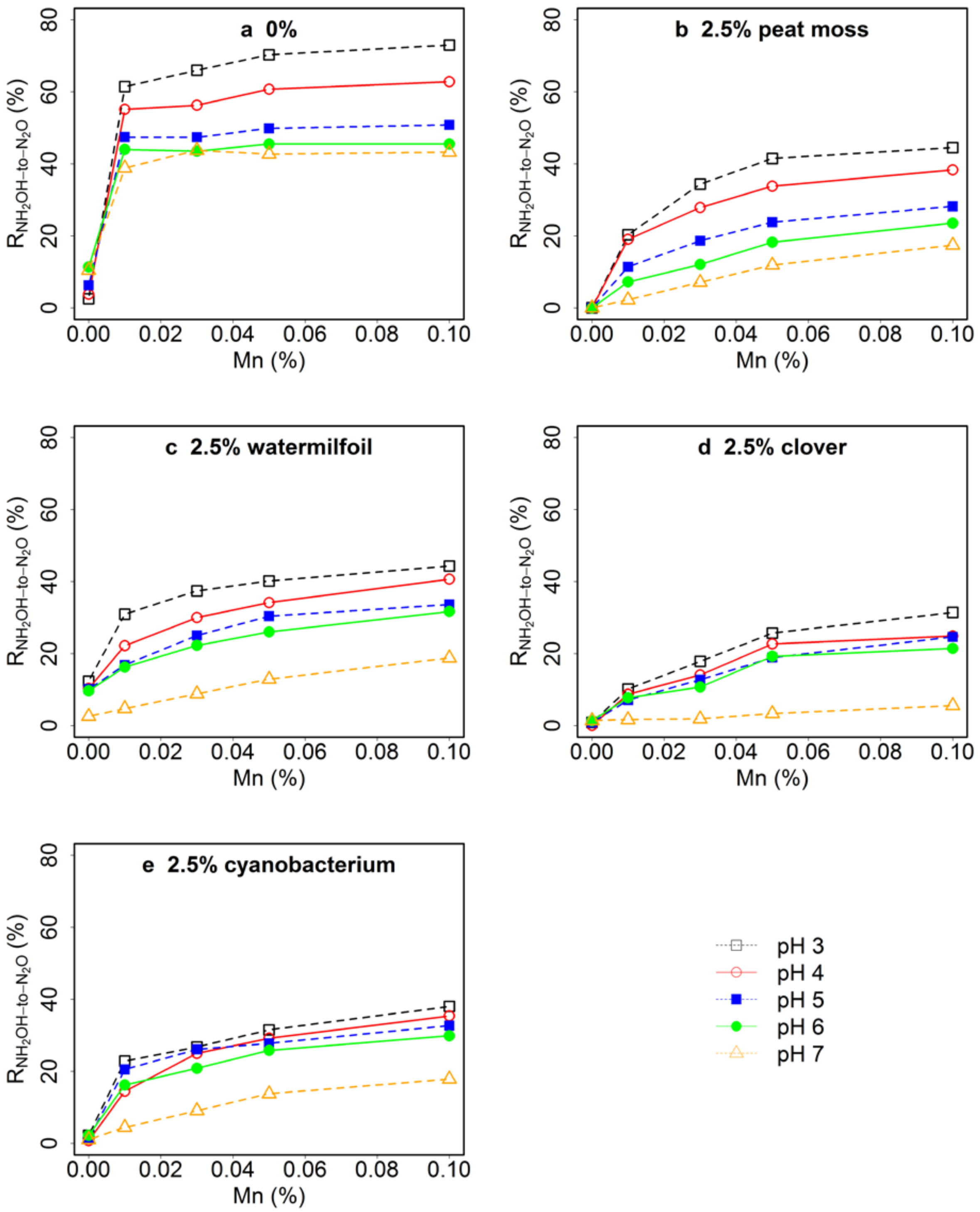

Figure S3 NH_2OH-to-N_2O conversion ratios ($R_{NH_2OH\text{-to-}N_2O}$) in artificial soils at different pH and MnO_2 content, and for organic matter (OM) of different origins at a fixed content of 2.5% (w/w). The total amount of NH_2OH added was 5 nmol. Different symbols represent $R_{NH_2OH\text{-to-}N_2O}$ for the artificial soil mixtures at different pH levels.

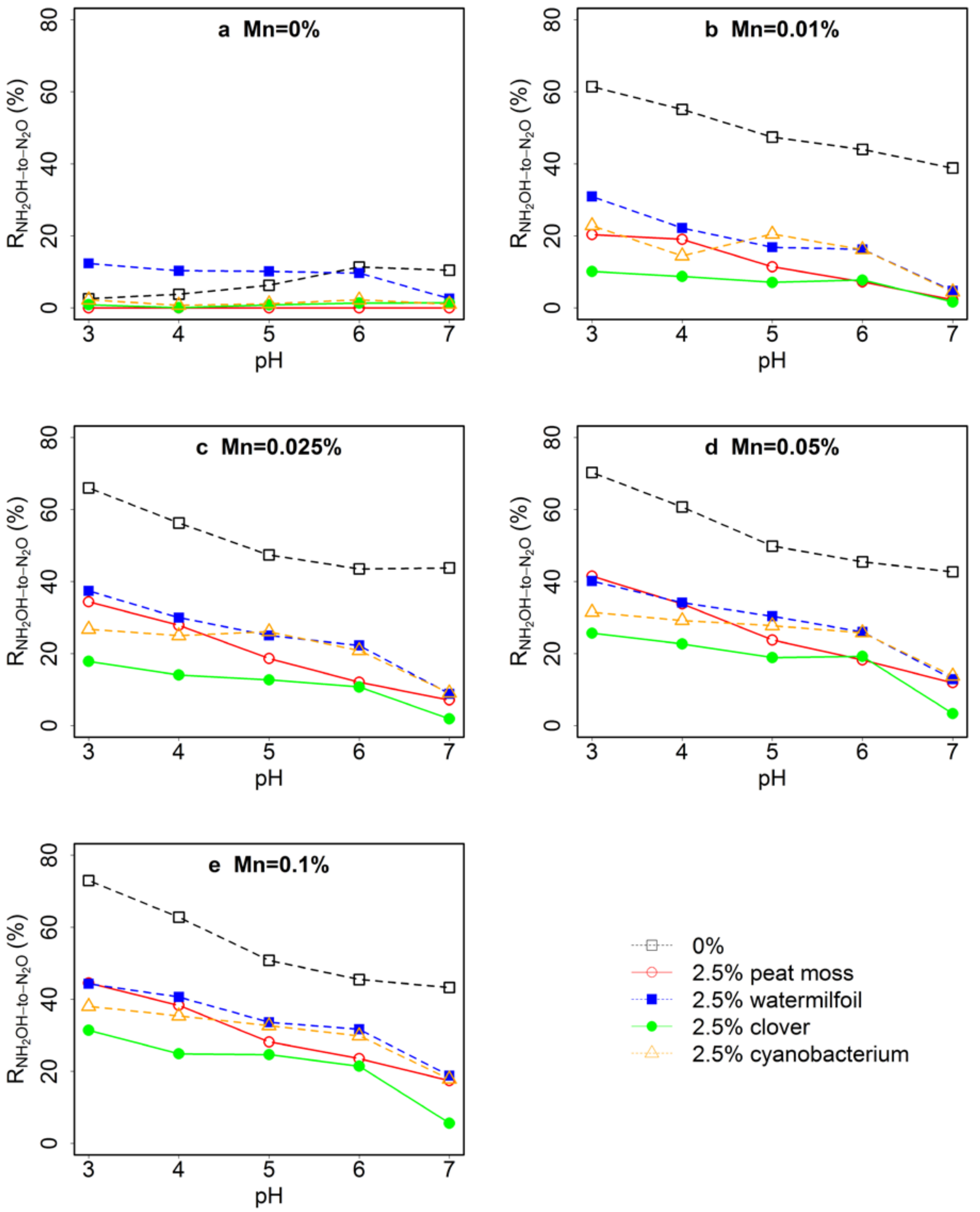

Figure S4 NH_2OH-to-N_2O conversion ratios ($R_{NH_2OH\text{-to-}N_2O}$) in artificial soils at different pH and MnO_2 content, and for organic matter (OM) of different origins at a fixed content of 2.5% (w/w). The total amount of NH_2OH added was 5 nmol. Different symbols represent $R_{NH_2OH\text{-to-}N_2O}$ for the artificial soil mixtures with different OM.
